# A Strategy for Alleviating Micro Arcing during HiPIMS Deposition of DLC Coatings

**DOI:** 10.3390/ma13051038

**Published:** 2020-02-26

**Authors:** Catalin Vitelaru, Anca Constantina Parau, Lidia Ruxandra Constantin, Adrian Emil Kiss, Alina Vladescu, Arcadie Sobetkii, Tomas Kubart

**Affiliations:** 1 National Institute of Research and Development for Optoelectronics—INOE 2000, 409 Atomistilor St., P.O. Box MG 05, 077125 Magurele-Bucharest, Romania; anca.parau@inoe.ro (A.C.P.); lidia.constantin@inoe.ro (L.R.C.); kadremil@yahoo.com (A.E.K.); alinava@inoe.ro (A.V.); 2 National Research Tomsk Polytechnic University, Lenin Avenue 43, 634050 Tomsk, Russia; 3 SC MGM STAR CONSTRUCT SRL, 7 Pancota St, 022773 Bucharest, Romania; sobetkii@yahoo.com; 4 Department of Electrical Engineering, The Ångström Laboratory, Uppsala University, P.O. Box 534, SE-751 21 Uppsala, Sweden; tomas.kubart@angstrom.uu.se

**Keywords:** magnetron sputtering, carbon sputtering, high power impulse magnetron sputtering (HiPIMS), process optimization, Ar+Ne+C_2_H_2_ gas mixture, diamond like carbon (DLC), mechanical properties

## Abstract

In this work, we investigate the use of high power impulse magnetron sputtering (HiPIMS) for the deposition of micrometer thick diamond like carbon (DLC) coatings on Si and steel substrates. The adhesion on both types of substrates is ensured with a simple Ti interlayer, while the energy of impinging ions is adjusted by using RF (Radio Frequency) biasing on the substrate at −100 V DC self-bias. Addition of acetylene to the working Ar+Ne atmosphere is investigated as an alternative to Ar sputtering, to improve process stability and coatings quality. Peak current is maintained constant, providing reliable comparison between different deposition conditions used in this study. The main advantages of adding acetylene to the Ar+Ne gas mixture are an increase of deposition rate by a factor of 2, when comparing to the Ar+Ne process. Moreover, a decrease of the number of surface defects, from ~40% surface defects coverage to ~1% is obtained, due to reduced arcing. The mechanical and tribological properties of the deposited DLC films remain comparable for all investigated gas compositions. Nanoindentation hardness of all coatings is in the range of 25 to 30 GPa, friction coefficient is between 0.05 and 0.1 and wear rate is in the range of 0.47 to 0.77 × 10^−6^ mm^3^ N^−1^m^−1^.

## 1. Introduction

Diamond-like carbon (DLC) coatings are widely used in many applications, due to their unique combination of properties such as high hardness, high density, low friction coefficient, chemical inertness etc. [1]. Although the deposition of DLC coatings has been investigated in many studies, it still represents scientific and technological open issues [2,3]. Different techniques were employed such as PECVD (Plasma Enhanced Chemical Vapour Deposition) [4], vacuum arc [5], pulsed laser deposition [6], magnetron sputtering [7,8] etc., depending on the required properties and the use of the coatings. The wide variety of achievable properties is mainly related to the bonding configuration of carbon, the sp^3^/sp^2^ ratio being a crucial parameter that defines the quality and potential use of the material.

One of the main factors determining the sp^3^ bonding in DLC is the ion bombardment, with an optimum energy around 100 eV for impinging C^+^ ions [9]. For the hydrogenated coatings in particular, where some of the carbon-carbon bonding can be converted into carbon-hydrogen, the bombardment with a sufficient number of ions that have the appropriate kinetic energy becomes even more important in increasing the fraction of sp^3^ bonds [10]. For the case of magnetron sputtering, significant ionization of sputtered atoms can be usually achieved by applying high power pulses, technique known as high power impulse magnetron sputtering (HiPIMS) [11,12,13,14]. The use of HiPIMS for the deposition of DLC coatings is a relatively new topic, with first reports in 2003 [15] and then explored, adapted and optimized in different variants [16,17,18,19]. In the case of C, the ionization degree is rather small even in HiPIMS, mainly due to the high ionization energy E_i_ of carbon (11.26 eV). This results in small C^+^ to Ar^+^ ratios, as low as ~1% of C^+^ from total ion contribution as shown in [14] and also low C^+^/C ratios in the deposition flux, as small as 5%, as shown in [15]. The ionization degree of C can be increased by increasing the electron temperature T_e_, that leads to a decrease of ionization mean free path [20]. One practical solution to increase the electron temperature is to change the working gas to one with higher ionization energy than the commonly used Ar (Ei = 15.76 eV). In this respect, Ne (E_i_ = 21.5 eV) was shown to be a good candidate for increasing T_e_ [18] and obtaining higher peak current values both to the target and to the substrate [21]. The use of Ne as sputtering gas was also investigated in a semi-industrial setup [22]. The maximum hardness of DLC coatings obtained using Ne was 45 GPa, significantly higher than the hardness of the coatings obtained using Ar, 25 GPa [22]. In more recent contributions it was also shown that the ionization degree of C can be increased by using positive pulses in the afterglow of HiPIMS [23,24].

The increased ionization provided by HiPIMS is beneficial also in reactive deposition of DLC. The use of a reactive environment by the addition of acetylene, to either Ar, Ar/Ne or Ne environment was demonstrated in obtaining hydrogenated DLC coatings with low hydrogen content, with hydrogen contents up to 15%–20% [10,25]. In that case, the HiPIMS plasma activates the reactive gas precursor. The high-density plasma creates radical ions and neutrals that contribute to the film growth, in addition to the C resulting from sputtering, giving rise to higher deposition rates at equivalent average injected power [25,26].

A common issue in C sputtering is the occurrence of micro-arcs. The frequency of arcing depends on the target material quality and its surface state during sputtering [27] and also on the peak current used for sputtering [21,28]. A stable operating regime can be established in a so called “mixed mode” sputtering [28,29], where a transition to arc occurs towards the end of the pulse and provides potentially higher current intensities and ionisation degrees. This was shown to be useful for obtaining highly tetrahedral amorphous carbon films [19], and studied as a separate version of HiPIMS when dealing with carbon sputtering. Nevertheless, the tendency for arcing and relatively difficult control of the process presents potential additional issues regarding the quality of the thin films, the formation of droplets on the growing film increasing the surface roughness. Since the increase of ionization degree and consequently of sp^3^ content can be achieved by increasing the peak current to the target above a certain critical level [21,30], the occurrence of uncontrolled micro-arcs becomes a limiting factor. Further increasing of the current and potential increase of sp^3^ content and hardness can come with unwanted higher density of defects on the surface.

In this contribution, we demonstrate a HiPIMS process for producing relatively thick, in the micrometer range, DLC coatings on Si and steel substrates. When comparing different process conditions it is very common to use the average power of the discharge as a constant parameter. In the case of HiPIMS on the other hand, it was previously shown that the peak current is the key parameter governing the sputtering regime and the ionization degree of the sputtered species [21,30]. We discuss therefore in the present paper, the use of the peak current as the main control parameter, to compare different deposition processes. Further, we propose the addition acetylene into the gas mixture to improve both the process stability and the quality of the coatings. Only a small amount is added, around 2% from total gas flow, so that the hydrogen incorporation to the forming film remains small.

## 2. Materials and Methods

The experimental setup consisted of a vacuum vessel equipped with five magnetron sources with circular targets of 50 mm diameter, mounted in a confocal geometry. In the present study only two of them were used, one made of C of 99.99% purity for HiPIMS sputtering of C and one made of Ti of 99.99% purity for DC sputtering of metallic interlayer. The C magnetron cathode was powered by a home-made HiPIMS power supply, providing stable voltage level throughout the pulse duration and operating at a frequency of 500 Hz and 30 μs pulse duration for all the experiments. The peak current was maintained at 25 A by adjusting the discharge voltage. More details about the process control are given in Section 3.1. The pulse characteristics were registered using a Lecroy 1:100 voltage probe and Tektronix TCP303 current probe with TCPA300 amplifier. The Ti magnetron was powered by a DC power supply DCXS-750-4, operating in a constant power mode at 105 W.

**Figure 1:**
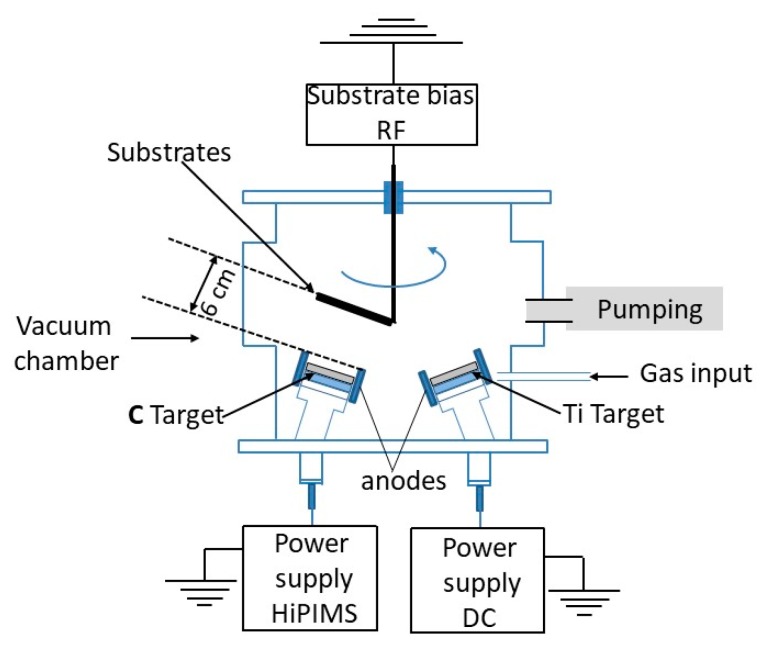
Experimental Setup.

A schematic representation of the experimental setup is presented in Figure 1. A movable substrate holder was located at a distance of 60 mm from the respective target surface. The holder was moved in front of Ti and C for interlayer and C deposition, respectively. Two types of substrates were used, namely 15 × 15 mm^2^ (100) Si samples, 500 mm thick, and 304 L steel discs 25 mm diameter and 1 mm thick. The substrates were sputter cleaned using an RF (Radio Frequency) discharge with 250 V of negative DC self-bias for 15 min. The substrates were biased during the deposition using RF biasing, with a DC self-bias of −100 V during C deposition, and at −50 V for Ti deposition. The biasing voltage at −100 V was chosen based on previous studies that showed an optimum around this values in terms of film density and hardness [9,22,28,31]. All samples were obtained at a total pressure of 2 Pa, maintained by an automatic valve that regulates the pumping section. Different mixtures of gasses were used, comprising Ar (99.9999% purity), Ne (99.999% purity) and C_2_H_2_ (99.6% purity). The substrates were not intentionally heated nor cooled during the deposition. The temperature of the substrate holder, as estimated by adhesive single use thermal sensors, was inferior to 125 °C on for all deposited samples.

The films were characterized by scanning electron microscopy (SEM), surface images were obtained by using a SEM-Tabletop Microscope TM3030PLUS (Hitachi, Tokyo, Japan) and cross section SEM images were obtained using a SEM/EDS/EBSD–Zeiss Merlin. The H content of the coatings deposited on Si samples was evaluated using ToF ERDA with 36 MeV I ions. Dektak-150 surface profiler was used to evaluate film thickness and to register the surface profiles. The mechanical characteristics of the coating were measured using the nanoindentation technique, performed with Hysitron TI Premier Nano indenter equipped with a Berkovich tip with a tip radius of 120 nm. The indentations were performed with a 3.5 mN indentation force, leading to maximum indentation depths lower than 80 nm and contact depths hc around 40 nm. The tribological properties were tested in a ball on disk configuration using a sapphire ball (6 mm diameter) in ambiental air, humidity in the 40% to 50% range and temperature ~22 °C. The tests were performed on circular wear tracks with radius of 5 mm at a constant load of 10 N, and for a distance of 400 m, corresponding to about 1270 cycles for each test. The tribological performance was evaluated based on friction coefficient and wear rate. The determination of wear rate was performed according to the method detailed in [32].

## 3. Results and Discussions

The results and discussions section is organized in three parts. The first one deals with the sputtering process, discussing the choice of deposition parameters. The second one is dedicated to the deposition and characterization of the DLC films on Si substrates with focus on the DLC mechanical and morphological properties, whilst the third part describes the mechanical and tribological characterization on metallic substrates.

### 3.1. Process Description

Sputtering of C under high peak currents as in the case of HiPIMS is a particularly challenging task, one of the main difficulties laying in the fact that micro-arcing can occur on the sputtered surface [28]. The proximity to an arc discharge can potentially provide advantages such as higher ionization degree, denser and harder coatings [33], but can also bring some of the disadvantages, like the micro-droplets formation on the growing film [22,28]. Therefore, in this work we focused on finding a stable process with discharge currents sufficiently high to achieve significant ionization of C while keeping the number of arcing events low. The main goal was to obtain hard coatings with low friction coefficients and wear rates, and with smooth surface. Two main approaches were followed and combined for tuning the process: (i) addition of Ne into the gas mixture, in order to improve the C ionization [18,34] and (ii) use of acetylene to increase the deposition rate through the contribution of C atoms from decomposition of gas precursor [10,35].

In this work, four different gas compositions (Table 1) were analysed and compared. Let us note that the externally adjusted process parameter was the discharge voltage, regulated by the power supply. The corresponding peak current and discharge power were determined by the plasma conditions, that in turn, were affected by other parameters such as gas composition, chamber geometry, magnetic field etc. In the reported process, change of the processing atmosphere by addition of either Ne or of C_2_H_2_ had a similar effect on the electrical behaviour of HiPIMS pulses. In both cases, an increase of the peak current at a constant discharge voltage was observed, in agreement with earlier reports [21]. The probability of arc ignition increased proportionally with the discharge current, as it was shown in [29], due to the creation of hot spots on the target that provided the seeds for arc formation. Therefore, a natural choice for reducing the number of arcs would be to maintain the same peak current as in the Ar process, while reducing the discharge voltage when the gas mixture is changed.

**Figure 2:**
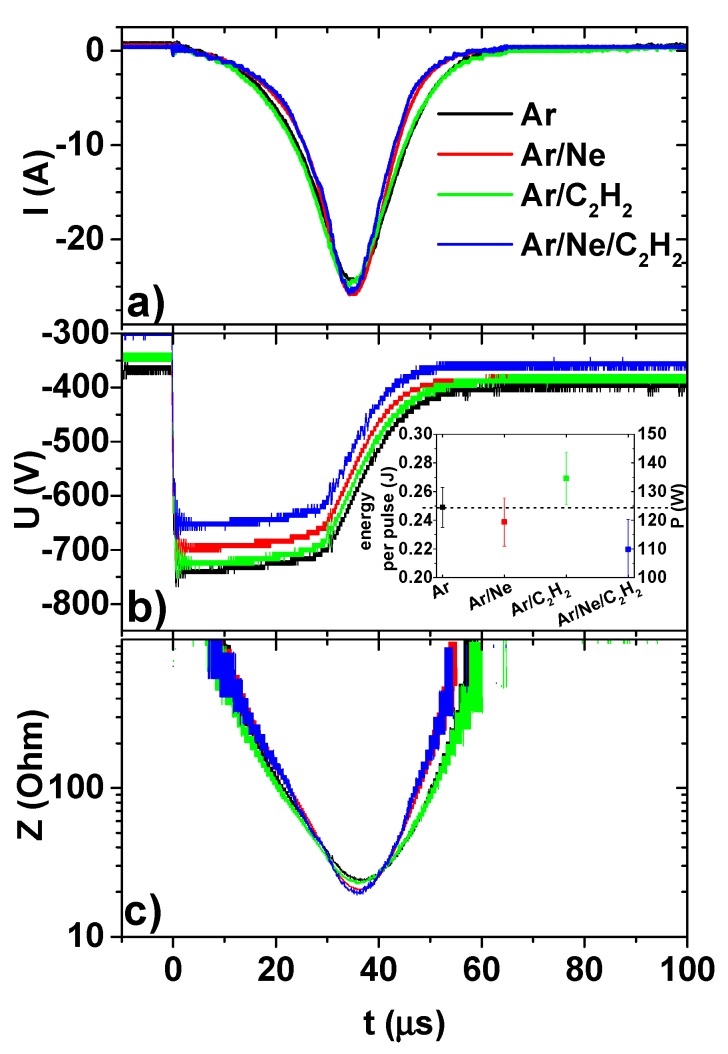
Typical time characteristics of current (I) (**a**), voltage (U) (**b**) and total impedance (Z) (**c**) during the pulse in different gas compositions. Average power P is in the 110 to 135 W interval, inset of (**b**).

Figure 2a,b shows typical current and voltage waveforms during the pulse, for gas compositions listed in Table 1, obtained by adjusting the voltage so that the peak current remains constant. It can be seen that the highest voltage was required in pure Ar, whilst for all other gas mixtures the voltages were lower, with a minimum for Ar/Ne/C_2_H_2_ mixture. The energy per pulse was calculated as the integral of instantaneous power time dependency during the pulse, and total power was calculated by multiplying the energy per pulse with the frequency of the pulses. The evolution of the two parameters is represented in the inset of Figure 2b. The values were obtained by averaging the results over at least 10 pulses. Another important parameter describing the sputtering regime is the minimum plasma impedance during the pulse, as it was previously described in our paper [21]. It was shown that the high current regime was characterized by low plasma impedance, on the order of 10–20 Ohm. Therefore, the plasma impedance for the investigated cases is represented in Figure 2c, showing indeed that the same high intensity sputtering regime was active for all cases. To summarize, the electrical characteristics of the pulses in Figure 2 show that same peak current was used, with comparable average powers in the range of 110 to 135 W, while sputtering in the same high current regime characterized by low plasma impedance.

### 3.2. Characterization Thin Film Deposited on Si Substrate

**Figure 3:**
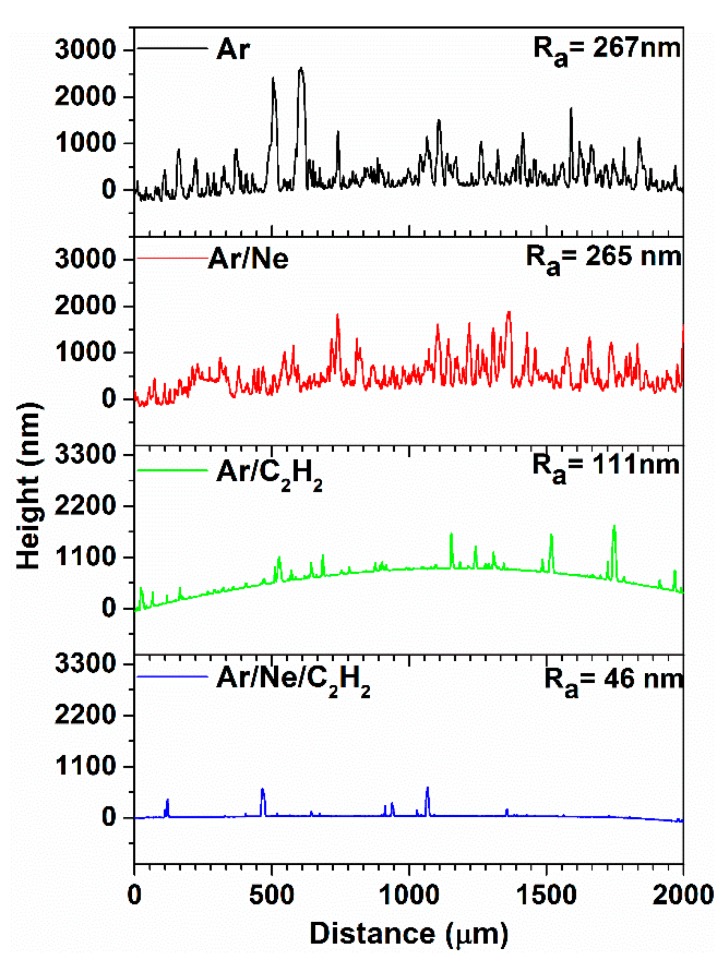
Typical roughness profiles of each coating prepared according to four process conditions presented in Table 1.

DLC films were deposited on Si substrates using all four sputtering conditions (Table 1). The typical surface profiles, chosen among a series of similar registered profiles, are displayed in Figure 3 for all four sputtering conditions. The average roughness Ra, as resulted from five different scans on 2 mm length, is also marked in Figure 3 for each deposition condition. It can be clearly seen that the Ar and Ar/Ne processes resulted in a rough surface, Ra > 250 nm, whereas the reactive process gives much smoother profile, Ra~50 nm, with only few features appearing. The smoother surface appears to be the one obtained by combining both Ne and C_2_H_2_.

**Figure 4:**
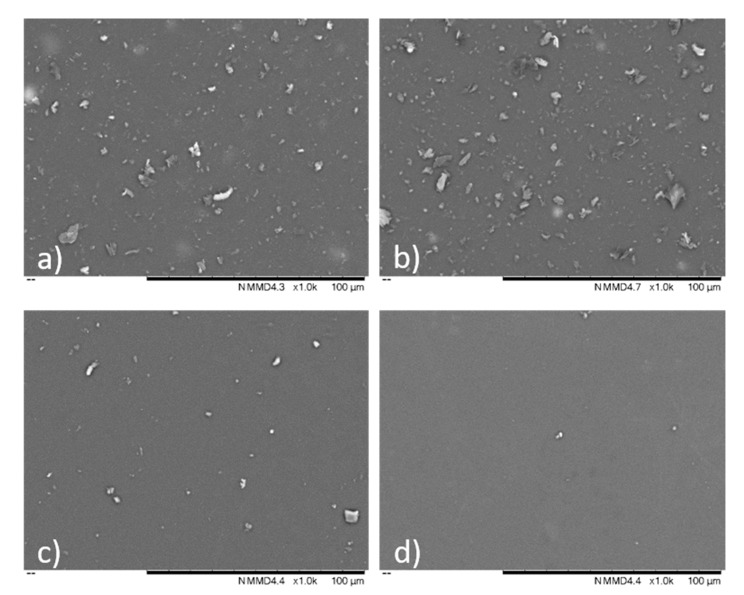
Typical SEM images of surface topography of coatings obtained in different gas atmospheres: (**a**) Ar, (**b**)Ar/Ne, (**c**) Ar/C_2_H_2_, (**d**) Ar/Ne/C_2_H_2_.

In order to have a better representation of the surface state, SEM imaging was performed. Four typical images are presented in Figure 4, showing the surface topography for each of the chosen sputtering conditions. It can be seen that the film roughness was associated with presence of surface droplets, which were denser on the surface of the coatings obtained in the Ar an Ar/Ne processes. Indeed, the DLC films appeared to have smooth surfaces with particle embedded in the surface, originating from the micro-arc events during the deposition process. The number of such particles was significantly lower for C_2_H_2_ containing gas mixtures, with the lowest number on the same unit area obtained in a Ar/Ne/C_2_H_2_ gas mixture. The percentage of area covered by dropplets was estimated by using Fiji imaging software [36], resulting in a 39% coverage for the coatings obtained in Ar/Ne process, compared with less than 1% are covered by dropplets for the coating obtained in A/Ne/C_2_H_2_ gas mixture.

**Figure 5:**
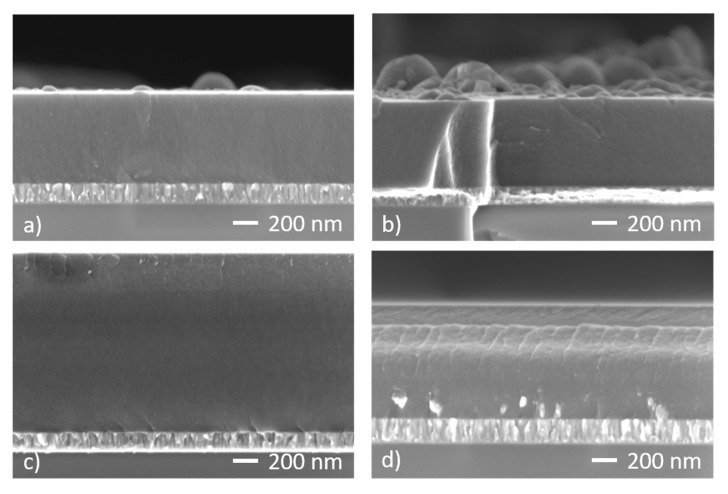
Typical cross section SEM on of coatings on Si substrates obtained in different gas atmospheres: (**a**) Ar, (**b**)Ar/Ne, (**c**) Ar/C_2_H_2_, (**d**) Ar/Ne/C_2_H_2_.

For more detailed analysis of the coatings microstructure, cross sectional SEM images were acquired (Figure 5). The differences in coatings thicknesses were related to different deposition rates and deposition times, as follows: coatings in Figure 5a,b were deposited for 120 min, coating in Figure 5c was deposited for the same duration of 120 min with C_2_H_2_ addition whereas coating in Figure 5d was obtained in only 70 min. Coatings c and d show comparable deposition rates, nearly twice higher than a and b that were sputtered without C_2_H_2_.

This increase in deposition rate is due to the contribution from C_2_H_2_ added to the gas mixture. It can be seen that in all cases the DLC coating presents a dense featureless microstructure, without any visible columns, characteristic for hard and dense DLC coatings. We can therefore conclude that the coatings keep their dense structure for all investigated conditions, with some features present for the hydrogenated coatings.

The surface defects, their distribution and density can be observed for the coating labelled Ar/Ne in Figure 5b, on the top surface of the coating. As can be seen these defects can have high density and sizes comparable to the film thickness. Therefore, it is important to find ways to avoid them, since these defects can influence the properties of the coatings and their behaviour in applications. Another aspect visible on all coatings is the Ti adhesion layer, with ~100 nm thickness and a columnar structure, characteristic to the DC process used for producing it. This very simple layer was proven to be sufficient to sustain DLC layers both on Si and 304 L steel, for thicknesses up to 1 μm.

**Figure 6:**
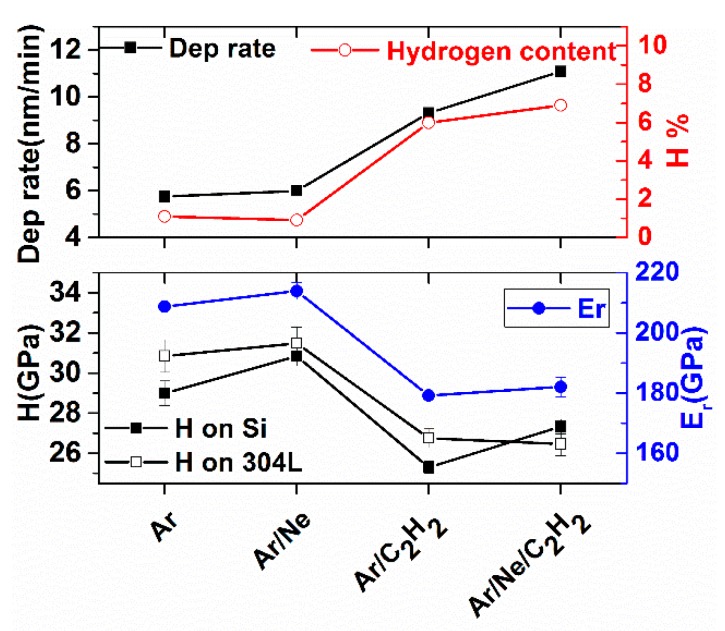
Deposition rate, hardness, reduced Young modulus and Hydrogen content variation as a function of deposition process.

One potential advantage of using a precursor gas is the increased deposition rate, due to the contribution of C atoms from the decomposition of C_2_H_2_. The HiPIMS plasma activates the C_2_H_2_ precursor promoting CVD carbon deposition, as it was shown in [10,25]. The deposition rate dependence on the sputtering process is represented in Figure 6, showing that the deposition rate almost doubles, from 6 to 11 nm/min, upon C_2_H_2_ addition. The introdcution of the gaseous precursor, however, leads to an incorporation of H in the film. The H content of the coatings measured by ERDA correlates with the deposition rate, Figure 6. The H concentration remained low, below 8%, but it could still have decreased the film hardness, as previously described in [25]. The hardness of films deposited on Si samples is also shown in Figure 6 as a function of deposition process. The non-reactive process resulted in less than 1% hydrogen incorporation and hardness around 31 GPa. The hardness decreased to 26–28 GPa upon C_2_H_2_ addition. This result indicates a trade-off regarding the hardness of the coatings, but a two fold increase of deposition rate can be a compelling argument in many industrial applications. Moreover, the hardness obtained for hydrogenated coatings was higher than the values found in the literature for comparable sputtering conditions, 7 to 19 GPa in [10] and 5 to 27 GPa in [35]. The reduced Young modulus variation measured on films deposited on Si substrate is also plotted in Figure 6, showing a similar trend with the one obtained for hardness, with ~210 GPa modulus for Ar and Ar/Ne processes and ~180 GPa for the hydrogenated coatings.

Another interesting aspect regarding the hardness variation is that the Ne addition, in a 50/50 gas mixture, did not increase significantly the hardness of the coating when compared to Ar plasma. This finding confirms the results previously described in [21] and attribute to the high peak current density of 2 A/cm^2^ used in this work. It was previosly shown that when using Ne only as sputtering gas the hardness of the coatings could be increased significantly, when compared to coatings obtained in Ar only [22]. It is to be noted that the peak power was increased by a factor of 1.5 when using Ne compared to Ar [22], making it difficult to fully separate the gas effect from the current increase effect.

### 3.3. Mechanical and Tribological Characterization of DLC on Metal Substrates

The same conditions presented in Table 1 were used for the deposition of DLC coatings on 304 L substrates, adjusting the deposition times such as the total thickness was ~1200 for all coatings. The hardness of the coatings was also determined for the coatings deposited on 304 L substrate, with the results represented in Figure 6 as a function of the deposition process. The same trend as for coatings on Si is observed, with lower hardness values for the reactively deposited coatings. This proves the reproducibility of the process, the coatings on Si and 304 L being deposited in separate processes.

**Figure 7:**
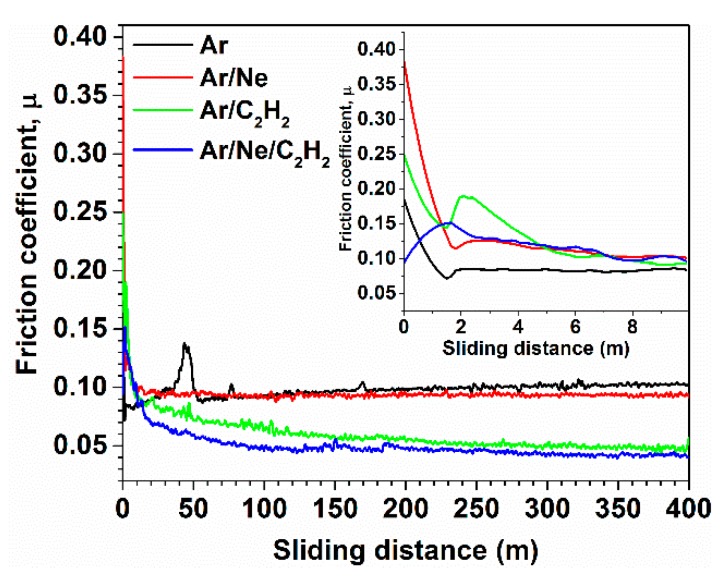
Friction coefficient variation on sliding distance of 400 m, under 10 N normal load, with sapphire ball counterpart versus coatings.

The coatings deposited on 304 L steel substrate were used for tribological characterization in the ball-on-disk tests. The evolution of the friction coefficient with sliding distance can be seen in Figure 7. After a very short running-in for a distance of about 2 m, as seen in the inset, the friction coefficient reached a steady value below 0.1 after only 10 m for all the coatings. The decrease can be attributed to the formation of a tribo-layer at the contact between the ball and the coating, a layer that contributes to the improvement of friction behaviour [37]. Once the tribo-layer gets formed the friction coefficient for the hydrogenated coatings reached a steady value of around 0.05 after about 150 m, whereas the hydrogen free coatings maintained a friction coefficient of 0.1. The values of friction coefficient are characteristic for hard C coatings [38]. The introduction of hydrogen is known to reduce the friction coefficient for DLC coatings [39].

**Figure 8:**
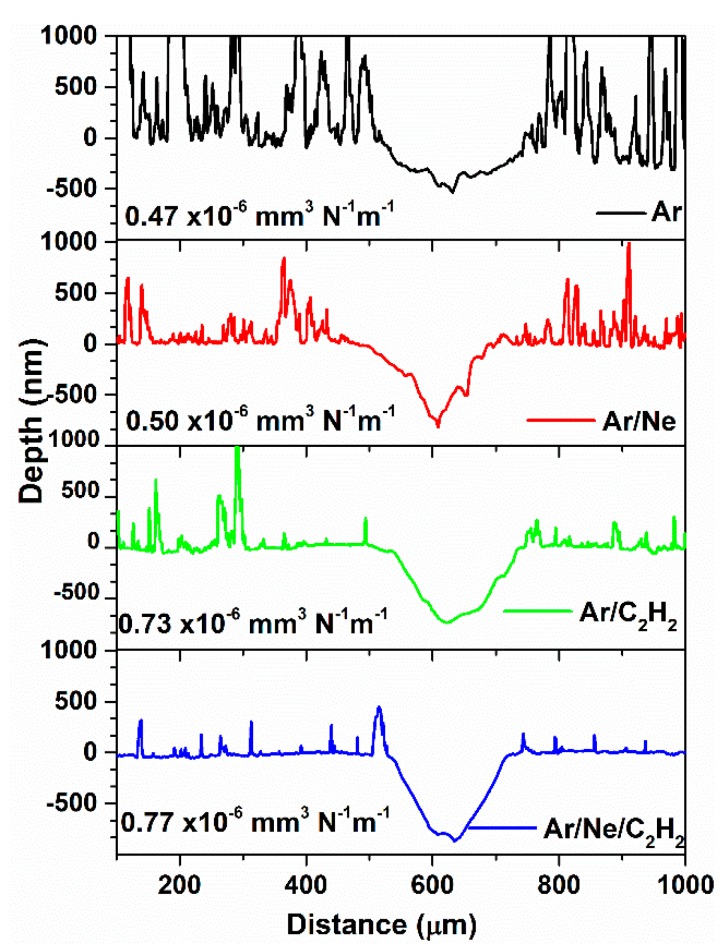
Wear track profiles on four coatings on 304 L steel, obtained in the conditions described in Table 1 and Figure 1.

The wear track profiles were measured by profilometry after 400 m of sliding. Typical profiles corresponding to the chosen process conditions are presented in Figure 8 along with the wear rate calculated as an average over four profiles. The wear of the hydrogenated coatings was only slightly higher than the H-free ones, as seen both from the depth of the wear profiles and the values of the wear rates. This was well correlated with the hardness values of the coatings, the hardest ones being also the ones that exhibited lower wear rate. There was a good correlation also with the microstructure of the coatings, the H-free one being slightly more compact and dense, as seen in Figure 5, having higher hardness and lower wear rate than the hydrogenated ones. The depth of the wear tracks did not exceed the thickness of the coating, which was ~1.1 μm for all the coatings deposited on 304 L steel. In all investigated cases the coating remained almost undamaged after the tribological testing, without any visible exfoliation marks.

**Figure 9:**
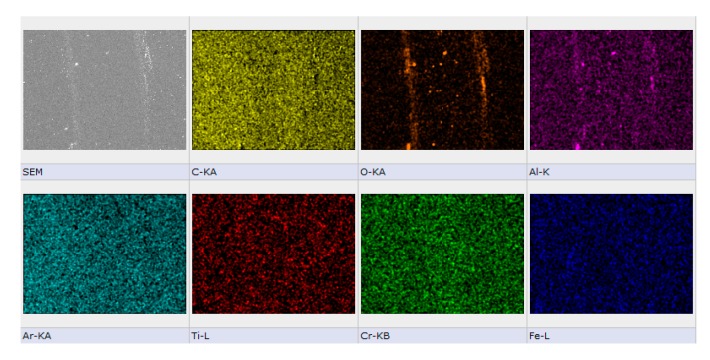
SEM-EDS images of the wear track on the coating obtained in Ar/C_2_H_2_ gas mixture after the tribological test with sapphire ball as counterpart. F = 10N, sliding distance 400 m.

As an example, a SEM-EDS image of the wear track is presented in Figure 9 for the sample Ar/C_2_H_2_. According to the SEM image of wear track, it can be seen that the C deposition seemed to be a bit polished after the tribological testing, the C layer remained intact inside the wear track. The debris that were visible on both sides of the race track originated from the sapphire ball, having a high content of Al an O, the constitutive elements of sapphire ball (Al_2_O_3_)_,_ as seen in the EDS mapping.

## 4. Conclusions

In summary, this contribution demonstrates tuning of the HiPIMS process for high quality DLC coatings with hardness in the range 25 to 30 GPa, both on Si and 304 L steel substrates. The adhesion of coatings up to 1mm thickness was ensured by using a Ti interlayer, deposited prior to DLC deposition without breaking the vacuum. The energy of the impinging ions was maintained constant by using an RF biasing unit that provided −100 V DC self bias during the DLC deposition and -50V during Ti interlayer deposition. It is shown that the addition of a small amount of C_2_H_2_ (~2% of the total gas flow) has a beneficial impact on the process stability at equivalent peak current. The observed reduction of arc events resulted in a significative reduction of surface defects, reducing the fraction of film surface covered by defects from ~40% to less than 1%. The effect on mechanical and tribological properties was small, with only about a 10% decrease of hardness and corresponding increase of wear rate. The activation of C_2_H_2_ in the high density plasma reduced the incorporation of hydrogen into the coatings with the H concentration below 7%. At the same time, the deposition rate was significantly increased by about a factor of 2. The improvement of surface roughness was accompanied by a decrease of friction coefficient from 0.1 to 0.05. The addition of acetylene, therefore, opens a useful process window for deposition of DLC with attractive properties at an increased deposition rate. The careful adjustment of the pulse parameters and the processing gas atmosphere reduces the frequency of arc events, enabling the use of higher peak currents with a reduced risk of surface defects formation.

## Figures and Tables

**Table 1:** Overview of the four deposition atmosphere mixtures used in this work, the pulse temporal characteristics and the peak current were kept constant.

Process Name	Ar (sccm)	Ne (sccm)	C_2_H_2_ (sccm)
**Ar**	**10**	**0**	**0**
**Ar/Ne**	**5**	**5**	**0**
**Ar/C_2_H_2_**	**10**	**0**	**0.2**
**Ar/Ne/C_2_H_2_**	**5**	**5**	**0.2**
